# Endocrine, auxological and metabolic profile in children and adolescents with Down syndrome: from infancy to the first steps into adult life

**DOI:** 10.3389/fendo.2024.1348397

**Published:** 2024-04-08

**Authors:** Silvia Molinari, Chiara Fossati, Maria Laura Nicolosi, Santo Di Marco, Martha Caterina Faraguna, Francesca Limido, Laura Ocello, Claudia Pellegrinelli, Martina Lattuada, Alessandra Gazzarri, Alessandra Lazzerotti, Debora Sala, Chiara Vimercati, Giulia Capitoli, Cecilia Daolio, Andrea Biondi, Adriana Balduzzi, Alessandro Cattoni

**Affiliations:** ^1^ Department of Pediatrics, Fondazione IRCCS San Gerardo dei Tintori, Monza, Italy; ^2^ School of Medicine and Surgery, University of Milano-Bicocca, Milano, Italy

**Keywords:** Down syndrome, hypothyroidism, hyperthyroidism, hypogonadism, diabetes mellitus, osteopenia, osteoporosis, obesity

## Abstract

Down syndrome (DS) is the most common chromosomal disorder worldwide. Along with intellectual disability, endocrine disorders represent a remarkable share of the morbidities experienced by children, adolescents and young adults with DS. Auxological parameters are plotted on syndrome-specific charts, as growth rates are reduced compared to healthy age- and gender-matched peers. Furthermore, children with DS are at increased risk for thyroid dysfunctions, diabetes mellitus, osteopenia and obesity compared to general population. Additionally, male individuals with DS often show infertility, while women tend to experience menopause at an overall younger age than healthy controls. Given the recent outstanding improvements in the care of severe DS-related comorbidities, infant mortality has dramatically decreased, with a current average life expectancy exceeding 60 years. Accordingly, the awareness of the specificities of DS in this field is pivotal to timely detect endocrine dysfunctions and to undertake a prompt dedicated treatment. Notably, best practices for the screening and monitoring of pediatric endocrine disorders in DS are still controversial. In addition, specific guidelines for the management of metabolic issues along the challenging period of transitioning from pediatric to adult health care are lacking. By performing a review of published literature, we highlighted the issues specifically involving children and adolescent with DS, aiming at providing clinicians with a detailed up-to-date overview of the endocrine, metabolic and auxological disorders in this selected population, with an additional focus on the management of patients in the critical phase of the transitioning from childhood to adult care.

## 1 Introduction

Trisomy 21 is the most frequent chromosomal aberration and the leading genetic cause of intellectual disability in the general population (1), with an incidence ranging from 1:700 to 1:1000 among live births and a prevalence of 1:400-1:3000 (2).

As a result of remarkable improvements in the care of affected children, adolescents and adults over the last decades, the scientific community has witnessed a dramatic increase in the life expectancy of people with Down syndrome (DS), currently set beyond the age of 60 years (3).

Accordingly, it’s pivotal that clinicians involved in the lifetime care of individuals with DS are provided with proper theoretical and practical expertise to face the progressively increasing burden of trisomy-related medical complications.

Thyroid disease, diabetes, obesity, short stature, subfertility and low bone mineral density account for a significant share of the medical complications recorded among subjects with DS.

By performing a comprehensive review of published literature, our objective was to provide clinicians with a detailed and updated overview of endocrine, metabolic, and auxological disorders recorded in children and adolescents with DS.

**Figure 1:**
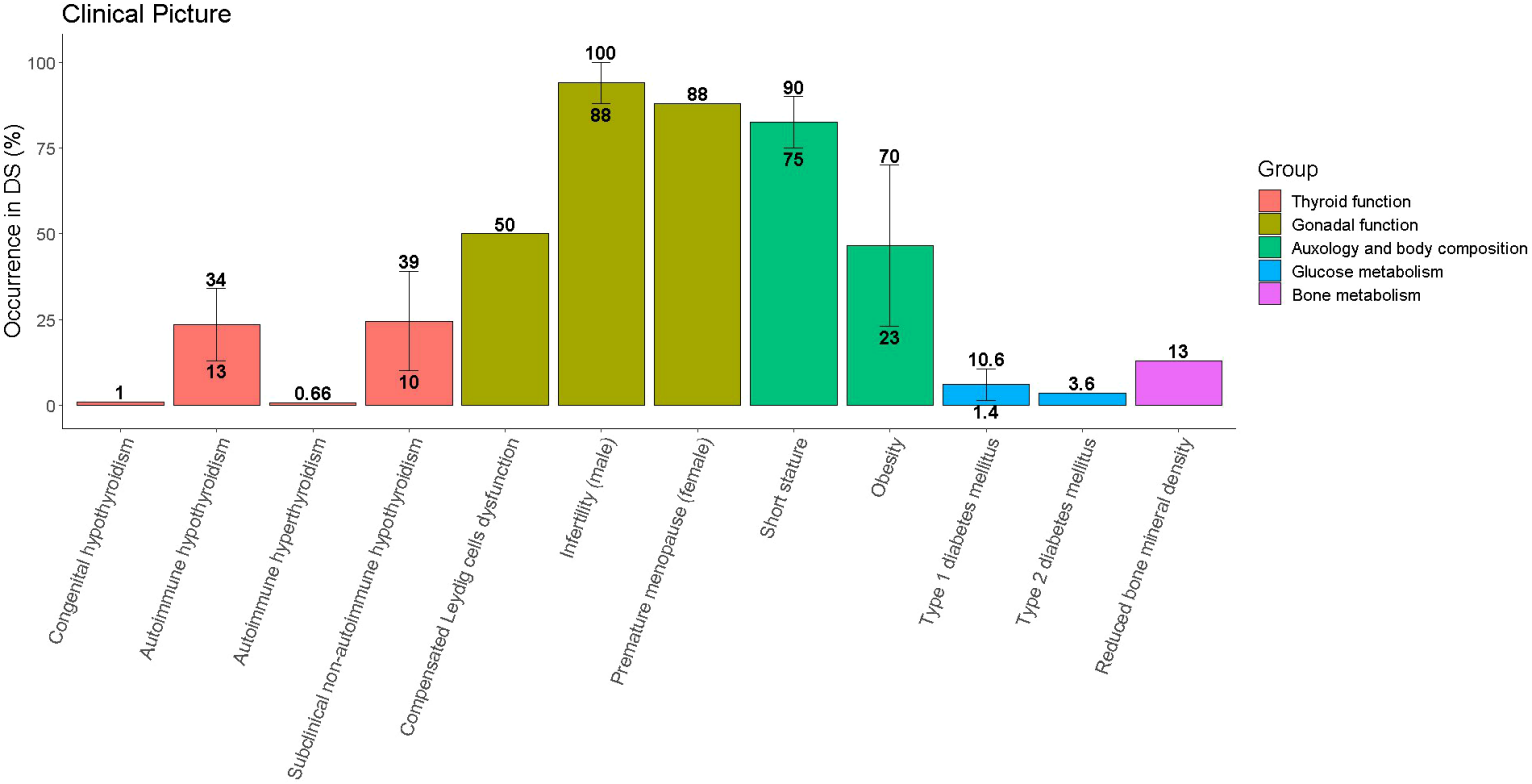
Occurrence of endocrine disorders among children and adolescents with Down syndrome (DS). Single disorders are grouped with reference to the organ involved. Whenever literature reports different prevalence for the same disorder the median value was represented by the height of the bar, while the range was represented by the vertical line connecting the lowest and the greatest occurrence.

**Table 1** and **Figure 1** provide a summary of the most frequently reported endocrine disorders among children and adolescents with DS, along with additional information about the specificities of each clinical picture in the setting of trisomy 21.

**Table 1:** Summary of endocrine, auxological and metabolic disorders potentially experienced by children and adolescents with Down syndrome (DS).

Class of endocrine disorders	Clinical picture	Occurrence in DS	Recommended screening to promote early detection in DS	Treatment in DS	Specificities of the condition in children and adolescents with DS
**Thyroid function**	Congenital hypothyroidism	1%	- Dry blood spot on Guthrie card for all newborns - TSH at the end of the neonatal period	L-thyroxine, to be started as soon as possible and always within 14 days of life	- Up to 30% of cases are transient - Anatomic findings: *in situ* gland with normal volume: 5-59%; gland hypoplasia 18-83%; goiter: 0-22%; agenetic or ectopic gland: 0-8%
	Autoimmune hypothyroidism	13-34%	- TSH at 6 months and 12 months of age and annually thereafter - Annual thyroid gland palpation and assessment of potential symptoms - Thyroid auto-antibodies in case of goiter or abnormal TFT	L-thyroxine	- Wide variability of effective L-thyroxine doses have been reported in DS (0.3 to 6.6 mcg/Kg daily) - Anti-TPO antibodies show a remarkably better positive predictive value than anti-TG
	Autoimmune hyperthyroidism	0.66%	- TSH at 6 months and 12 months of age and annually thereafter - Annual thyroid gland palpation and assessment of potential symptoms - Thyroid auto-antibodies in case of goiter or abnormal TFT	-Anti-thyroid medications; - thyroidectomy -radioiodine therapy	- Prolonged administration of anti-thyroid medications should be pursued, given the greater occurrence of adverse effects of surgery (anatomic features) and radioiodine treatment (risk of secondary malignancies) in DS subjects
	Subclinical non-autoimmune hypothyroidism	10-39%	TSH at 6 months and 12 months of age and annually thereafter	L-thyroxine	-In asymptomatic individuals, TSH threshold above which treatment is recommended is still controversial - slight upward shift of average TSH levels in DS compared to healthy controls have been reported - In over 70% of cases, TFT normalize within 5 years
**Gonadal function**	Compensated Leydig cells dysfunction (male)	Marginally increased LH with normal testosterone reported in up to 50% of Tanner 5 subjects	No specific monitoring is recommended, as testosterone levels are commonly normal	None	Adolescents with DS often experience increased LH levels in the setting of normal testosterone levels (compensated hypergonadotropic hypogonadism)
	Infertility (male)	Increased FSH levels have been reported in up to 88-100% of post-pubertal adolescents with DS	No specific monitoring is recommended	None	Adults with DS are generally regarded as infertile, though three cases of spontaneous parenthood have been reported
	Premature menopause (female)	Up to 88% of women with DS aged 46 to 50 years are in menopause	Complete assessment of gonadal function in case of early-onset secondary amenorrhea	Hormonal replacement therapy if required	Female individuals with DS are regarded as potentially fertile, but experience a precocious decrease in oocyte reserve, thus leading to menopause earlier than healthy controls (median age: 46 years *versus* 51-52)
**Auxology and body composition**	Obesity	Prevalence: 23-70% in childhood, 50% in adulthood	Weight gain and BMI should be assessed at least yearly upon clinical periodic evaluations	Dietary intervention, calories management and physical exercise should be promptly recommended in case of progressive weight gain	People with DS show lower lean body mass and an excess of visceral fat compared to age-, gender- and BMI-matched healthy controls
	Short stature	The height of 75-90% of adults with DS falls below the 3^rd^ centile with reference to the general population growth charts	Height attainment in children and adolescents with DS should be assessed by syndrome-specific growth charts	Recombinant human growth hormone is not routinely recommended in individuals with DS	Average final height in subjects with DS is 153 cm in males and 143 cm in females
**Glucose metabolism**	Type 1 diabetes mellitus	Prevalence: 1.4-10.6%	- Caregivers should be periodically informed about signs and symptoms potentially consistent with hyperglycemia, in order to prompt timely diagnosis and treatment - Fasting blood glucose and HbA1c levels should be assessed whenever the clinical suspicion of type 1 diabetes mellitus is raised	Insulin	- Children and adolescents with intellectual disability, as in DS, particularly benefit from the introduction of sensors for continuous glucose monitoring instead of self-monitoring blood glucose by fingerstick - Treatment-wise, recent technologic advances play a pivotal role in achieving a satisfactory glycemic control in patients with intellectual disability and in preventing them from experiencing severe or frequent episodes of hypoglycemia
	Type 2 diabetes mellitus	Prevalence: 3.6%	- Fasting blood glucose and HbA1c levels should be assessed in all children with DS aged 10 years or more with age- and gender-matched BMI above the 85^th^ centile or presenting specific risk factors (maternal history of gestational diabetes, family history of T2DM, high-risk ethnicities, signs of insulin resistance) - Among non-obese asymptomatic individuals with DS, screening for T2DM (fasting glucose and HbA1c levels) should be assessed at the age of 30 years and every three years thereafter	Oral antidiabetic medications or insulin administration based on metabolic control, as per international guidelines for otherwise healthy patients	Children and adolescents with intellectual disability, as in DS, particularly benefit from the introduction of sensors for continuous glucose monitoring instead of self-monitoring blood glucose by fingerstick
**Bone metabolism**	Reduced bone mineral density	From 13% in adolescence and young adulthood, to 66% in elderly individuals.	Systematic screening by DEXA scan is not recommended in pediatrics. CAYA with DS who experience fractures should undergo a biochemical evaluation to rule out potential underlying causes (hyperthyroidism, celiac disease, vitamin D deficiency, hyperparathyroidism, the use of detrimental medications) and a DEXA scan	The potential benefits of bisphosphonates in treating low bone mineral density in subjects with DS is still debated	Diminished osteoblastic activity and inadequate bone mass accrual, rather than abnormalities in bone resorption, are responsible for the low bone mass observed in individuals with DS. Accordingly, the potential efficacy of antiresorptive therapies is debated

CAYA, children, adolescents and young adults; DS, Down syndrome; TFT, thyroid function tests; TPO, thyroid peroxidase; TG, thyroglobulin; BMI, body mass index; LH, luteinizing hormone; FSH, follicle stimulating hormone; T2DM, Type 2 diabetes mellitus; TSH, thyroid stimulating hormone; DEXA, Dual energy X-ray absorptiometry.

## 2 Thyroid function

Thyroid disorders are more frequent in children with DS than in the general population, with an age-related prevalence ranging from 4-8% in children to 54% in adults. Individuals with DS experience a 6-fold greater occurrence of hypothyroidism than hyperthyroidism. In addition, in patients with DS more commonly than in healthy controls, autoimmune dysthyroidism encompasses a dynamic spectrum of disorders, with recurrent shifts from hypo- to hyperfunctioning gland dysfunctions and vice versa (4, 5).

Firstly, children with DS are prone to develop congenital hypothyroidism (CH) compared to healthy controls (6). The first mainstay of prompt detection and treatment of CH is the Newborn Screening (NBS) program by dry blood spot test (7). The negative impact of a misdiagnosed congenital hypothyroidism on cognitive development is well-known; as a variable degree of developmental delay is typical of DS, early diagnosis of hypothyroidism in newborns with DS is pivotal (8, 9).

In addition, children with trisomy 21 present a higher risk of developing autoimmune disorders, probably due to syndrome-related dysregulation of immune tolerance (10). Therefore, both Hashimoto thyroiditis and Graves’ disease are highly frequent in this population. Epidemiological studies report that anti-thyroid antibodies are found in 1.3% of otherwise healthy children *versus* 13-14% of people with DS, highlighting a 10-fold greater occurrence of autoimmune thyroid diseases (11).

In the following paragraphs we will provide detailed information about the specificities of each thyroid disorder in the population of pediatric individuals with DS.

### 2.1 Congenital hypothyroidism

CH, defined as an increase of Thyroid Stimulating Hormone (TSH) values with low/normal fT4 and fT3, is usually diagnosed in the first weeks of life through the NBS programs. The overall reported incidence of CH in DS is about 1% in Western Countries, 28 times greater than in healthy children (12).

In this sub-population, gland hypoplasia, which can already be identified during fetal stage, is the most frequent abnormality (18-83%) (13). On the other hand, partial or total thyroid agenesia, as well as ectopic gland or goiter are overall rare findings among newborns with CH and DS (14, 15). Furthermore, in 5-59% of cases, ultrasound reveals an *in situ* gland without any dimensional or morphological defect (12).

The significant occurrence of CH in the setting of anatomically normal glands has raised questions about the pathophysiology of congenital impaired thyroid function in DS. Luton et al., by autoptic analyses on 13 fetuses with DS, highlighted a paucity of follicles, supporting the hypothesis of a less effective hormonal stimulus (13).

The overexpression of dual-specificity tyrosine-(Y)-phosphorylation regulated kinase 1A (DYRK1A) gene, located on chromosome 21, has been identified as a potential pathogenic factor, playing a detrimental role on thyroid follicle maturation from the fetal stage onwards (16).

In addition, the incidence of transitory CH is greater among newborns with DS than in healthy controls. Indeed, in up to 30% of patients, thyroid function normalizes over time (17). It is likely that the abovementioned sub-optimal gland function may become overt during periods of increased metabolism, such as the neonatal period, concomitant malformations (e.g. cardiopathy), or perinatal complications. Whenever newborns with DS meet the criteria for substitutive treatment with L-thyroxine, treatment is commenced, following the same indications provided for otherwise healthy infants. Nevertheless, after the first three years of life, when hormone-dependent development of the central nervous system is fulfilled, endocrinologists should consider attempting therapy discontinuation, especially whenever concomitant complications that may have been negatively affecting thyroid function over the neonatal stage have been addressed and effectively treated.

Positive predictors of successful L-thyroxine withdrawal are persistently normal TSH values, lower TSH values at diagnosis and stable doses of levothyroxine over time (18).

Therapy goals are the same as those for any child with CH: to achieve TSH within age- and kit-specific reference ranges, tolerating increased fT4 levels as long as not associated with TSH suppression (19). Notably, in newborns and infants with DS and congenital heart disease, increased FT4 levels should prompt a strict biochemical and clinical follow-up, that could eventually lead to a reduction of L-thyroxine dose, in order to prevent children from experiencing cardiac arrythmias and acute complications.

According to European Reference Network recommendations for the management of CH, treatment must be started as soon as possible, before the age of 14 days of life, with an initial dosage of L-thyroxine of 10-15 µg/kg/day. Higher doses (15 µg/kg/day) are indicated in severe cases (fT4<5pmol/L) and lower doses (10 µg/kg/day) in milder cases (19).

Given the high prevalence of CH in DS, in case of a normal neonatal screening, frequent monitoring throughout the first year of life is recommended. The American Academy of Pediatrics (AAP) recommends evaluation of thyroid function at least three times during the first months (at birth, 6 and 12 months), and annually afterwards (20). Most recently, the European Reference Network guidelines for the management of CH in infants with DS recommend one additional evaluation at 1 month of life (birth-1-6-12 months and then annually) (19). Other Authors, such as Pierce et al., endorse thyroid function control at 6-8 weeks of life and at 4 months (21). These additional screening timepoints are recommended due to the high occurrence of neonatal comorbidities in DS. Indeed, affected newborns experience a high prevalence of non-thyroidal illness as a consequence of cardiac or intestinal disease. Accordingly, TSH generation may be impaired, resulting in a false-negative neonatal screening. Additional measurement of TSH and fT4 in the first weeks/months of life may prompt the timely detection of undiagnosed CH in this selected sub-cohort of patients.

### 2.2 Autoimmune hypothyroidism

The incidence of Hashimoto thyroiditis is higher in individuals with DS compared to the general population (10, 22). In this setting, anti-thyroid peroxidase (TPO) antibodies show a better positive predictive value compared to anti-TG, especially in terms of unfavorable evolution from subclinical hypothyroidism/euthyroidism to overt gland hypofunction (23, 24).

The gene *AIRE*, autoimmune regulator, is located on chromosome 21 and codifies for a master controller of immune tolerance, regulating the expression of tissue-specific antigens in the thymus and T-cells selection. Its function, as well as that of other genes, is reduced in individuals with DS due to the trisomic imbalance, and is associated to thymic hypofunction, resulting in a higher incidence of autoimmune disorders (25, 26). Such pathogenesis explains the lack of gender polarization among patients with DS and autoimmune thyroid disorders, in contrast with the remarkable prevalence in females within the general population, for whom hormonal factors play a pivotal role.

Hypothyroidism in children with DS occurs at an earlier age compared to healthy peers, but such finding may be the result of systematic screening programs, leading to more frequent testing among asymptomatic subjects (5, 12). Diagnosis of Hashimoto thyroiditis more frequently precedes or follows the onset of extra-thyroid autoimmune diseases, such as alopecia (prevalence 6-10%), celiac disease (5-10%) or diabetes mellitus type 1 (3 times more frequent than in the general population) (5, 11).

Diagnosis is more often made by routine blood testing, rather than by clinical findings, as hypothyroidism in children with DS is less symptomatic or presents with a specific signs such as hypotonia and weight gain, which are common in people with trisomy 21 regardless of thyroid function (12, 27–29).

Patients with Hashimoto thyroiditis and overt hypothyroidism (low FT4 and compensatory increased of TSH) are candidate to replacement therapy with L-thyroxine. Conversely, replacement therapy is not recommended in asymptomatic patients with normal thyroid function tests, though a systematic follow-up should be undertaken in order to timely detect eventual progression into hypothyroidism. Finally, several factors should considered in the decisional process that eventually leads to prescribe L-thyroxine in patients with subclinical hypothyroidism, such as TSH values, reported symptoms and the clinical or sonographic finding of goiter (10, 30).

Levothyroxine dosage is titrated, until biochemical euthyroidism is achieved. Though an average dosage of 1-2 mcg/kg/day is usually sufficient in the general population, daily doses ranging from 0.3 to 6.6 mcg/kg have been reported in children and adolescents with DS (31). The first evaluation of efficacy by assessing thyroid function tests is indicated not earlier than 6 to 8 weeks following treatment prescription and then every six months (32).

### 2.3 Autoimmune hyperthyroidism

Graves’ Disease shows prevalence rates as high as 0.66% in children and adolescents with DS, thirty folds greater than in the general population (33). As for Hashimoto disease, the pivotal role of genetics on the pathogenesis of thyroid disorders results in an earlier disease onset and lack of gender polarization among children with DS (33).

In contrast with autoimmune hypothyroidism, hyperthyroidism more frequently determines an acute presentation with classical symptoms (palpitations, anxiety, heat intolerance, fatigue, tremors, increased appetite, weight loss) and signs (hyperactivity, tachycardia, arrhythmia, systolic hypertension, diaphoresis, hyperreflexia, muscle weakness). Accordingly, diagnosis is more frequently clinically-guided, rather than performed by routine laboratory testing (33, 34). Even so, the intellectual disability and subsequent difficulty in reporting symptoms experienced by patients with trisomy 21 may result in diagnostic delay in comparison to the general population.

Anti-TSH receptor antibodies and thyroid ultrasound should be sought whenever TSH is suppressed, either in the setting of a symptomatic patient or in case of occasional finding upon yearly monitoring.

There are three mainstay treatments: anti-thyroid medications such as methimazole, thyroidectomy and radioiodine therapy (33).

As for the general population, pharmacological treatment is the first therapeutic choice (35). Historically, several authors suggested that surgery should be considered in case of failure of remission after 2 years of treatment. Nevertheless, more recently, a growing body of literature has shed light on the beneficial effects of prolonged methimazole administration, if tolerated, at the minimum maintenance dosage before attempting treatment discontinuation, particularly in children (36–38).

Graves’ disease seems to have a more favorable course in individuals with DS compared to the general population. Indeed, the reported percentages of relapse following a first course of anti-thyroid medication are lower than in healthy controls (39). In addition, patients with DS show greater percentages of persistent remission over time and average lower doses of methimazole are generally needed to achieve biochemical euthyroidism in comparison to the general population (39, 40). Nevertheless, some Authors have challenged these results, thus supporting the hypothesis of a remarkable inter-individual variability in response, remission rates and dosages required (33).

Thyroidectomy represents second line treatment in otherwise healthy patients. Nevertheless, in children with Down syndrome, anatomic factors such as short neck and airway malacia, along with generalized hypotonia, result in a remarkable increase of anesthesiologic risk class, thus leading clinicians to provide indication to neck surgery only in case of demonstrated and unequivocal failure of medical approach.

Finally, radiohyodine administration results in gland function suppression, that prompts the need for a lifetime replacement therapy with levothyroxine (33). Importantly, exposure to radiation represents an independent risk factor for onset of hematologic malignancies (41) and it is therefore regarded as potentially harmful for subjects with DS, who are genetically predisposed to develop lymphoblastic and myeloid leukemias *per se*.

### 2.4 Subclinical and overt non autoimmune hypothyroidism

Subclinical non autoimmune hypothyroidism (SNAH) is defined as the finding of an isolated increase in TSH with normal free circulating thyroid hormones, in the absence of autoimmune disease, goiter or clinical symptoms.

The reported prevalence rates in people with DS is as high as 10-39% (12, 21, 42–44), thus resulting as the most frequent thyroid dysfunction in this population. In addition, it is likely that the real-life occurrence of SNAH is probably underestimated because of the asymptomatic nature of the disorder (31). Most of the remarkable variability in the prevalence range reported by different studies depends on the poor consensus about the gender- and age-specific reference ranges of TSH values in individuals with DS.

We recently assessed thyroid function tests over time among 550 pediatric children and adolescents with DS and outlined DS-specific reference ranges and nomograms for thyroid function parameters. Our data show a life-long upward shift of TSH compared to healthy age- and gender-matched peers (45). It has been hypothesized that the additional chromosome 21 may affect the regulation of hypothalamic-pituitary-thyroid axis, thus resulting in slightly increased average TSH levels compared to healthy controls.

The pathogenesis of subclinical hypothyroidism in subjects with DS may be multifactorial. Several determinants have been hypothesized by different authors as potentially involved in the pathogenesis of SNAH, such as delay of maturation of the hypothalamus-hypophysis-thyroid axis, inappropriate central secretion of TSH, lower biological activity of TSH, lower sensibility of TSH-receptor, inadequate dopaminergic down-regulation of TSH secretion and over-expression of genes located on chromosome (34, 46).

From a prognostic perspective, factors predicting the evolution of subclinical hypothyroidism into overt pictures are poorly reproducible. Thyroid function tests show a remarkable intra-individual variability over time, as proven in longitudinal long-term assessments (45). Accordingly, several authors have demonstrated that SNAH shows a transitory course in more than 70% of cases (29, 46), especially when TSH values are just above the upper reference limit (17).

Indications for starting thyroid hormonal replacement therapy in individuals with DS and SNAH are object of debate; to date, conflicting positions lead to a lack of consensus. The aim of levothyroxine would be to normalize TSH values, determining an improvement in developmental and cognitive performances. Van Trotsenburg et al. found a slight motor delay in 2-year-old infants with DS and subclinical hypothyroidism who were not treated in comparison to treated controls (19). Such difference was not observed over a ten-year long follow-up (47). Some studies revealed benefits of replacement therapy and normalization of TSH values in terms of growth velocity, height, and head circumference (17, 47, 48); no improvement of motor and cognitive development was found (47). Besides, most of these results have been systematically challenged by different authors, who did not confirm such findings (31).

In conclusion, efficacy of treatment has not been proved and there is no consensus on cut-off values for starting substitutive therapy. Nevertheless, most guidelines agree on treating patients with TSH values above 10μU/ml, goiter, or overt symptoms (10, 49). Before starting any treatment, thyroid function must be re-evaluated, given the remarkable interindividual variability (45).

### 2.5 Thyroid disorders: from adolescence to adulthood

In adults with DS, thyroid disorders represent the most common chronic comorbidities. The reported prevalence of hypothyroidism in this population ranges from 39% between 18 and 29 years to 51% in individuals aged 30 or older (50, 51).

Only few studies on the long-term evolution of thyroid function in adults have been published. In detail, conflicting outcomes have been reported regarding the advantage of treating elevated thyrotropin levels in asymptomatic patients, the diagnostic accuracy of antithyroid antibodies and the clinical utility of routinely assessing anti-thyroid antibodies to screen for thyroid disease in adults with DS (52).

Prasher and colleagues have demonstrated, through a long-term follow-up, that most individuals with DS develop thyroid disorders in childhood and adolescence, while over 73% of young adults without thyroid dysfunctions persistently show normal thyroid function tests as they grow older. In addition, most adult subjects with subclinical hypothyroidism are not expected to develop overt hypothyroidism (53).

Thus, infancy, adolescence and young adulthood are key phases for the overall life-long risk of developing dysthyroidism in DS. Nevertheless, also the guidelines for the management of adult patients with DS recommend regular screening for thyroid function tests every 1-2 years (52).

## 3 Puberty and gonadal function

While there have been numerous reports on gonadal dysfunction in individuals with DS, research on pubertal attainment and sexual development in this population is scarce and mostly limited to historical studies (54, 55). In addition, overall outcomes have been conflicting, with some studies reporting an increased incidence of gonadal dysfunction (54), while others demonstrating an intact pituitary-gonadal axis and retained levels of circulating sex hormones (55–57).

Concerning pubertal timing, historical analyses have outlined that the average onset puberty was set around 13 and 12 years in male and female people with DS, respectively (54, 57). These data apparently differ from those drawn by more recent studies. For example, in a cohort of children with DS, Erdoğan and colleagues reported pubertal onset at a median age of 10.0 years for both genders, which is superimposable with the outcomes more recently described for age-matched peers (58). In addition, the average age of menarche in adolescents with DS is consistent with the general population in several study cohorts (58–60). Overall, the difference in the timing of pubertal attainment reported by different authors likely mirrors most of the changes experienced by the general pediatric population over the last decades. Indeed, the tempo of pubertal onset and progression is influenced by multiple factors, including nutrition, genetics and the environment. Over the past century, the average timing of pubertal development has decreased by approximately 0.3 years every 10 years in the general population (61). This phenomenon has been attributed to improved nutrition and higher socioeconomic status among children and involves both otherwise healthy and syndromic people.

Due to the scarcity of data concerning gonadal function in pediatric subjects with DS, it is currently not possible to provide detailed information about the incidence of hypogonadism.

The most extensive studies on gonadal function held in DS have focused on male, post-pubertal individuals. Indeed, few analyses have assessed luteinizing hormone (LH), follicle-stimulating hormone (FSH), and testosterone levels among subjects with DS, aiming at reporting the prevalence of hypogonadism in this population (54, 56). Approximately 70% of the individuals enrolled displayed elevated FSH and LH levels compared to the median values observed in post-pubescent controls, while testosterone levels were found to be superimposable with the average values recorded in otherwise healthy adult males (56–58). Based on these biochemical criteria, a non-unremarkable share of people with DS could be classified as presenting “compensated hypogonadism” (high LH levels in the setting of normal total testosterone levels). These data support the hypothesis of a certain degree of Leydig cell impairment in DS and has also been described in early infancy. It could be inferred that this condition is most likely congenital in nature and, consistently, strictly related to trisomy itself (62).

The increase in serum FSH levels after puberty has been regarded as an indication of germ cell dysfunction, as reported in previous studies (54, 56). Accordingly, although three cases of spontaneous conception have been described (63–65), male individuals with DS are known for presenting with a significantly impaired fertility. On the one hand, this could be the mere result of the relatively small proportion of sexually active individuals with DS aiming at procreating. On the other hand, impaired spermatogenesis along with dysfunction in Sertoli cells among men with DS is likely to play a pivotal role (54, 61, 62). These hypothesis is also consistent with the average testicular volume recorded in subjects with DS, remarkably lower than those recorded in age-matched healthy controls (54, 61).

Conflicting data have been published concerning gonadal function among women with DS. Though no systematical association with primary ovarian insufficiency has been published, in some studies FSH levels were found to be abnormally high (54, 56). Conversely, Angelopoulou and colleagues described levels of gonadotropins within normal range (60). However, the average LH levels were significantly greater that those assessed in controls, consistently with the findings reported by other authors (54, 56).

Overall, women with DS have been shown to be potentially fertile, as highlighted by several reported cases of maternity (66–70). Nevertheless, in a post-mortem autoptic study conducted on girls with DS, ovaries were found to show either a lack of follicle maturation or delayed follicle development. Additionally, there were differences in the number and size of antral follicles, as well as an early decline in small follicles, particularly noticeable after the third year of life (71). These features remarkably diverged from the findings collected in otherwise healthy controls, who exhibited distinct patterns of follicle growth and development (71). Consistently, from a biochemical perspective, women with DS experience an early and significant decrease in anti-Müllerian hormone (AMH) levels, universally regarded as a marker of ovarian reserve and strictly related to the overall pool of ovarian antral-phase follicles (72). As a consequence, woman with DS tend to experience menopause at a marginally younger age compared to the general population (71–73), with a reported median age of 46 years.

## 4 Auxology

In children with DS, it is recommended that weight and height trends are monitored and plotted on syndrome-specific charts (74). Indeed, length/height, head circumference and growth velocity are lower from birth onwards, compared to age- and gender-matched non-trisomic peers.

### 4.1 Growth

Short stature is one of the main auxological features of Down Syndrome. The reported average adult height is 153 cm in males and 143 cm in females (75). Short stature is mostly due to reduced length of limbs, while the trunk commonly shows a retained size (75). Overall, sitting height-to-height ratio is greater compared to the general pediatric population.

The etiology of short stature in DS has not yet been completely clarified. Recent studies have reported a certain degree of impairment of the GHRH-GH-IGF1 axis in subjects with trisomy 21. In detail, some authors have highlighted that short stature in DS can result from a combination of unsatisfactory hypothalamus–pituitary secretion and reduced bioactivity of endogenous GH (76).

The prescription of recombinant human growth hormone (rhGH) in this population has been historically widely debated, due to ethical considerations on the benefits provided in terms of gain of height and due to conflicting results on its efficacy. The recurrent lack of controls, the limited follow-up, as well as the heterogeneous outcomes among treated patients has led to poorly reproducible conclusions (77). A recent meta-analysis reports a short-term efficacy of rhGH treatment on the growth of children with DS, irrespectively of endogenous GH secretion patterns (76). Nevertheless, most studies did not manage to demonstrate a longer-lasting and durable positive effect of the treatment on growth attainment (78–80). In addition, long-term analyses are lacking and systematic data about rhGH safety in a population exposed to a greater occurrence of malignant hematological disorders are not available. Accordingly, though DS patients should not be precluded from GH therapy *per se*, no clear indication can be provided and rhGH is often proscribed to date. Benefits and disadvantages should be discussed with the parents and child and the approach should be patient-tailored (76).

### 4.2 Body composition

With regard to body composition, infants present with poor weight gain from birth to infancy, mostly because of feeding-related issues due to hypotonia and macroglossia or associated comorbidities, including heart defects and gastrointestinal malformations (81). This trend tends to reverse from late childhood to adolescence, with reported obesity rates as high as 23-70% (82–85). Obesity can complicate conditions such as obstructive sleep apnea, diabetes and cardiovascular disease (86).

The cause of the increased occurrence of obesity and overweight must be sought in slow basal metabolism, low circulating leptin levels, hypotonia, susceptibility to systemic inflammation and possibly concomitant hypothyroidism (81, 83, 87, 88). In addition, impaired mobility and motor clumsiness may negatively affect physical activity and thus result in an overall sedentary lifestyle. Furthermore, several studies have connected the shortening of the telomeres, which causes accelerated aging in DS, with the increase of body mass index (BMI) and adiposity (89).

It is important that families are periodically advised about healthy diet programs and exercise promotion in order to prevent obesity (86). Pediatric endocrinologists and pediatricians play a pivotal role in preventing children and adolescents from gaining excessive weight and becoming obese adults. Indeed, obesity remains a leading clinical issue among adults with DS, with reported prevalence rates as high as 50%. Accordingly, a life-long follow-up with yearly weight and BMI check is strictly recommended among adults with DS. Dietary intervention, calories management and physical exercise are the main therapeutic measures, with the aim of reducing obesity-related comorbidities (86). Bariatric surgery is controversial in adults with intellectual disability (88).

## 5 Glucose metabolism

Though the scientific community has historically focused on the clinical and epidemiological burden of type 1 diabetes mellitus among children with DS, a growing body of literature has shed light upon the greater occurrence rates of type 2 diabetes compared to the general population (90).

### 5.1 Type 1 diabetes mellitus

The remarkable increase in the risk of developing autoimmune disorders experienced by individuals with DS (40, 91) is overt when looking at the four-fold greater prevalence of type 1 diabetes mellitus (T1DM) compared to the general pediatric population (90).

The first epidemiological report of this clinically significant association dates back to 1968, when Milunsky and colleagues firstly highlighted the occurrence of diabetes (either insulin-dependent or -independent) in a wide population of over 20.000 individuals diagnosed with trisomy 21 (92).

The median age at diagnosis shows a biphasic distribution, with a first peak before the age of 2 years and the second in early adolescence (10 years) (93). The median age for the onset of T1DM in children with DS is 8 years, about 6 years earlier than in the general pediatric population (94). Both genders show superimposable incidence rates (91).

The specificities of the epidemiology of T1DM in subjects with DS is strictly related to the abovementioned predisposition to develop dysimmune phenomena related to the over-expression of genes located on trisomic chromosome 21. Accordingly, children with DS experience an increased co-occurrence of type 1 diabetes and different autoimmune disorders, such as Hashimoto thyroiditis (74%), coeliac disease (14%) or both (8%), as reported by Aitken and colleagues (95).

Several genomic analyses have reported an excess of diabetes-associated HLA class II genotypes in children with both DS and T1DM compared to healthy controls (95). Nevertheless, DS children with T1DM are less likely to carry the highest- risk genotypes (i.e. DR4-DQ8/DR3-DQ2), as they are more prone to carry low-risk ones (95–97). Accordingly, it has been speculated that additional factors, possibly involving genes located on chromosome 21, may increase the penetrance of T1DM in children with DS. For example, the co-occurrence of increased copies and point mutations on both AIRE gene (21q22.3) and its promoter, may lead to the overexpression of its transcript, which regulates T-cell function and self-recognition, thus contributing to the secretion of anti-pancreatic islets autoantibodies (98).

Consistently, recent analyses have demonstrated a certain degree of chronic flogosis and over-expression of pro-inflammatory cytokines among individuals with DS, as a result of the constitutional dysregulation of IL-6 signaling pathway (99). This unfavorable biological *milieu* is known to promote autoimmune phenomena, with subsequent overproduction of autoreactive antibodies. All these features may contribute to the development of a breach into immune tolerance.

The clinical signs and symptoms of diabetes in children with DS are similar to those reported in the general pediatric population, but the younger age upon diagnosis in combination with the cognitive and language impairment related to the syndrome may result in a diagnostic delay (100, 101).

Despite the cited constitutional dysregulation of immune system, children with DS affected by T1DM seem to show an overall better metabolic control, requiring lower insulin daily dose, in comparison to age-matched peers from general population (96). Accordingly, patients with DS present a lower prevalence of diabetic complications (such as retinopathy, nephropathy and neuropathy). A potential explanatory hypothesis is that patients with DS often adopt a more systematic lifestyle and display an overall better compliance to routine treatment than otherwise healthy peers (102).

### 5.2 Type 2 diabetes mellitus

Concerning type 2 diabetes mellitus (T2DM), prevalence rates as high as 3.6% have been reported in children and adolescents with DS (103). As for the adult general population, higher body mass index (BMI), family history consistent with impaired glucose metabolism and female gender are all potentially detrimental factors playing a negative role upon insulin-sensitivity (96).

A recent study reported a 10-fold increase in the incidence of T2DM among individuals with DS aged 5 to 14 years compared to age- and gender-matched peers, while occurrence rates were only doubled over the age of 54 years (104).

This increased incidence is likely to be related to predisposition to peripheral insulin resistance and declining β-cell function as a consequence of obesity and reduced physical activity (105). Indeed, though fat mass index, percentage of body fat, and lean BMI are superimposable in adolescents with and without DS, syndromic individuals show lower lean body mass for a given BMI Z score compared to general population. In addition, visceral fat represents a greater share of the body fat mass recorded in subjects with DS (106). Overall, a less favorable body composition leads to greater occurrence rates of insulin resistance among trisomic patients.

### 5.3 The screening of glucose metabolism

As debated above, children and adolescents with DS show an increased risk of developing either type 1 or type 2 diabetes. Though no univocal guidelines about screening and treatment of impaired glucose metabolism in the pediatric population with Down syndrome have been published to date, several *consortia* have provided dedicated recommendations.

Timely detection and treatment of diabetes mellitus are crucial in this at-risk population. National and international *consortia* for the care of children and adolescents with intellectual disability recommend systematic assessment of signs and symptoms potentially consistent with hyperglycemia and prompt dedicated testing in case of clinical suspicion, given the 5-fold increase in the risk of developing DM recorded among patients with variable degrees of intellectual disability (100, 101). In addition, adequate information about signs and symptoms of T1DM should be provided to the caregivers of children with DS, in order to promote prompt diagnosis and early treatment (107). Despite a greater occurrence of T1- and T2DM in this selected cohort, there is poor agreement about the best screening approach to monitor glycemic metabolism. The AAP guidelines for the management of children and adolescents with DS do not recommend periodical systematical assessment of either blood glucose or glycated hemoglobin (HbA1c) levels among asymptomatic subjects (20). Consistently, the guidelines for the care of adults with DS advocate that screening for DM is started from the age of 30 years onwards and every 3 years thereafter (52). On the other hand, a growing body of independent charities focused on the care of children with DS has started to promote annual screening of fasting blood glucose levels and HbA1c from the age of 14 onwards (108).

Overall, clinicians should also remember to apply the recommendations provided by the American Diabetes Association (ADA) for otherwise healthy pediatric subjects. Indeed, screening for prediabetes and for T2DM is warranted in all asymptomatic children and adolescents aged 10 years or older with age- and gender-matched BMI above 85^th^ percentile or with at least one of the following risk for diabetes: maternal history of gestational diabetes and/or family history of T2DM among first or second degree relatives; or high-risk ethnicities (Native American, African American, Latino, Asian American, Pacific Islander); or signs of insulin resistance; or additional disorders associated with insulin resistance (109). The screening should be performed both with fasting glucose plasma level and HbA1c levels, every three years or more in case of rapidly increasing BMI. Oral glucose tolerance test should be prescribed for a more accurate evaluation of dysglycemia, such as when the clinical suspicion for diabetes or impaired glucose tolerance remains high despite non-diagnostic blood glucose or HbA1c levels (109). Similar recommendations can be drawn also for adult subjects with DS. Indeed, the ADA recommends that individuals showing a BMI of 25 or greater and at least one additional risk factor should undergo screening for type 2 diabetes every 3 years after achieving puberty (109). As DS can be regarded as an independent risk factor for T2DM, a dedicated biochemical screening should be undertaken in any overweight adult with DS, regardless of the presence of additional comorbidities (52).

Interestingly, children with DS tend to present with higher fasting glucose levels than otherwise healthy peers, but unexpectedly lower HbA1c levels (106). The reason for decreased HbA1c levels among participants with DS still needs to be clarified. Wachtel and Pueschel reported macrocytosis in individuals with DS as well as increased red blood cell turnover (110). If the latter is confirmed, a shortened red blood cell life span could lead to a factitious decrease in HbA1c levels.

### 5.4 Monitoring and treatment of diabetes mellitus

Children with T1DM and their families are generally encouraged to check blood glucose levels about four times a day, in order to customize insulin treatment and to avoid events of hyper- or hypoglycemia. In addition, these data provide healthcare professionals with an adequate amount of data to adapt insulin posology upon periodical evaluations.

In the last decades, the scientific Community has witnessed a dramatic improvement in the technological achievements for the monitoring of blood glucose levels among diabetic patients (111). Children and adolescents with intellectual disability, as in DS, particularly benefit from the introduction of sensors for continuous glucose monitoring (CGM) instead of self-monitoring blood glucose by fingerstick. Real-time CGM systems perform a systematic and continuous assessment of patient’s interstitial glucose levels over time. This technology is effective in providing the user with prompt information about current glucose reading, at any time of day. In addition, CGM devices generate patterns of glucose trends that can be analyzed retrospectively to clarify the overall glycemic balance, playing a pivotal role on potential patient-tailored therapeutic decisions. In addition, alarmed systems for the early detection of hypoglycemic episodes have remarkably improved the quality of life and reduced adverse events in this population of patients, that may be less aware of the signs and symptoms consistent with low blood glucose levels or less prompt in informing the caregiver about incipient severe hypoglycemia (112).

Treatment-wise, the technological innovations in insulin therapy (such as smart insulin pens and insulin pumps with automatic insulin infusion) play a pivotal role, nowadays, in achieving and maintaining satisfying glycemic control with a lower risk of micro- or macroangiopathic complications, in children with T1DM and DS.

Insulin pumps provide small amount of insulin and can be programmed to vary on an hour-by-hour basis. They are especially recommended in younger children and for those in whom hypoglycemia cause remarkable management issues (113).

Prevention of type 2 diabetes requires multifactorial treatment including lifestyle modification, regular physical exercise and body weight control. Metformin has been historically regarded as a mainstay in the treatment of T2DM in pediatrics. The use of liraglutide, a GLP1 agonist, has been recently approved both for weight reduction and for diabetes, also in the pediatric population. Obviously, insulin therapy is the first therapeutic choice in case of severe glycemic decompensation at the onset (111).

## 6 Bone health

The progressively increasing life expectancy in individuals with DS has shed light on the occurrence of osteopenia, osteoporosis and fractures, overall greater than the one recorded in the general population (114). Indeed, among adults with DS, about one out of four is affected by osteoporosis (115) and median bone mineral density (BMD) values recorded in this population are lower than in age- and gender-matched controls, irrespectively of the degree of intellectual disabilities (ID) (116, 117). Longitudinal studies have indicated that bone mineral density (BMD) decreases at a faster rate with age in individuals with DS compared to the general population (50), exposing adults with DS at an increased risk of developing osteoporosis and experiencing bone fractures (115). As DS is regarded as a progeroid syndrome (118), i.e. adults with DS present healthy issues common in elderly people, it could be hypothesized that the greater incidence of low BMD is the expression of an earlier occurrence of a disorder typical of aging (50). In addition, as previously discussed, the early onset of menopause in women with DS may play an additional detrimental role on bone health (115). Consistently, the evidence of a lower BMD has been found even in children and adolescent with DS, compared with healthy peers (119–121).

Dual-energy X-ray absorptiometry (DXA) is the gold standard to screen bone health, but people with DS are less likely to receive BMD testing, for several reasons including intellectual disability, that makes the procedure less affordable (115).

Additionally, DXA-derived BMD does not take into account the differences in bone size between DS and otherwise healthy controls, potentially overestimating osteopenia (116). Accordingly, volumetric BMD (vBMD or bone mineral apparent density (BMAD)) estimated on the three-dimensional parameter, should be preferred as it better reflects the skeletal assessment in subjects with DS, overcoming the problem of morphological differences (116). Moreover, adjusting BMD for body height, weight and total lean mass is even more important in children, in order to take into account their physical modifications due to growth (122). For these reasons, to date, only few studies on adjusted BMD have been conducted within the pediatric population with DS.

In a cornerstone study conducted by Carfi and colleagues, DXA was used to assess BMD at the femoral neck and lumbar spine in 234 adults with DS compared to 2206 adults from the general population, enrolled in the National Health and Nutrition Examination Survey dataset. The results revealed significantly lower mean BMD and BMAD in the DS group compared to controls. These findings also highlighted a strong association between these measurements and age, especially in the DS group. In this study, average BMAD levels of adults with DS aged 40–49 was superimposable to that of controls aged 60–69, suggesting the importance of initiating early screening for osteoporosis in adults with DS (50).

Interestingly, despite the reported universal agreement about the overall reduced median BMD among adults and young adults with DS (57, 60, 116, 120), conflicting opinions have been reported about bone density in children, mainly due to divergent methodologies, analyses, and sample features among published studies (116, 119, 121, 123). In detail, Wu and colleagues reported lower BMD and bone mineral content (BMC) exclusively in the pelvic region of 7-to-10 years-boys (123). Combining these results with those by González-Agüero and colleagues (119) and Baptista et al. (116), it can be speculated that in DS reduced BMD does not occur before adolescence and that the pelvis shows the earliest involvement (123). Concerning fractures, data drawn from the DS-Connect questionnaire showed that the occurrence of fractures in the enrolled population was as high as 27%, mostly involving younger age classes (124).

Concerning sex-driven prevalence, in contrast to the higher prevalence of osteoporosis and osteopenia in men with DS compared to women (121), female children and adolescents with DS are described to have a lower bone mass peak than age-matched trisomic males (122).

Data about bone turnover markers in the pediatric population with DS are lacking. In adults with DS, McKelvey and colleagues demonstrated that both women and men with trisomy 21, without consistent clinical risk factors for osteoporosis, showed lower levels of bone deposition markers compared to individuals without DS (125). This profile is significantly associated with low BMD, regardless of gender. These findings suggest that diminished osteoblastic bone formation and inadequate bone mass accrual are the main responsible for the low bone mass observed in individuals with DS. This contrasts with postmenopausal or senile osteoporosis, in which increased bone resorption is regarded as the primary pathogenic factor. These observations, confirmed also by more recent analyses (126, 127), raise doubts about the effectiveness of antiresorptive therapies in this specific population.

The etiology of osteoporosis in DS is multifactorial. The genetic imbalance due to trisomy 21 has been described as a main determinant of low bone mineral density in individuals with DS (114). Blazek et al. reported that transgenic Ts65Dn mice, owing 3 copies of about half of the genes of human chromosome 21, showed craniofacial and appendicular skeletal phenotype resembling the one observed in humans with DS (128). Subsequently, the same Authors described that the overexpression of DYRK1A gene induces skeletal abnormalities and abnormal bone homeostasis, especially in transgenic male mice, while a normal copy number of DYRK1A results in a normal development of the appendicular skeleton and bone turnover (129). In addition, utilizing a mouse mapping panel, it has been demonstrated that additional triplicated orthologous genes located on chromosome 21 may play a role in determining an abnormal skeletal phenotype (130). Also epigenetic modifications, e.g. overexpression of the receptor interacting protein 140 (RIP140) have been related to poorer bone health and may play a role in low BMD of people with DS (131, 132).

Besides genetics, several potential environmental risk factors for low BMD are common among patients with disability, such as poor dietary habits with scarce calcium and vitamin D intakes, limited sun exposure, sedentary lifestyle and reduced physical activity (120). Additionally, hypotonia and the frequent use of anticonvulsants may contribute to bone depletion (133), as well as the increased risk of developing coeliac disease and the forementioned endocrine disorders (45, 57, 103).

Preventive strategies include adequate calcium and vitamin D intake, appropriate sunlight exposure and the medical treatment of endocrine comorbidities (134). Similarly, routine physical exercise and active lifestyle are crucial to increase muscular strength and bone mass peak, especially during adolescence (119, 134–136).

Treatment options for osteoporosis in DS are currently limited and further studies are needed. Indeed, bisphosphonates may not provide benefits as they reduce bone turnover, instead of acting on bone accrual, which is described to be the main issue in patients with DS, as previously stated (125). Accordingly, Fowler and colleagues reported that PTH intermittent treatment in Ts65Dn mice significantly increased bone mass and volume suggesting that anabolic treatments, as intermittent-PTH and anti-sclerostin antibodies (127), may be effective in improving BMD in subjects with DS (136).

Finally, given the key role of genetics on bone health in DS, gene therapy, e.g. targeting DYRK1A, and histone manipulation for epigenetic modifications may be promising future solutions (114, 137).

## 7 Conclusions

In conclusion, DS is associated to a remarkable increase in the risk of developing multiorgan comorbidities, including endocrine disorders.

Thyroid disease represents a wide share among the endocrine disorders recorded in children and adolescents with DS. Given the remarkable occurrence of congenital and early-onset hypothyroidism, a strict systematic monitoring of thyroid function tests is warranted at birth and along the first year of life. Subsequently, the risk of developing autoimmune thyroid disorders prompts the universal indication of a lifelong screening program for thyroid health among subjects with DS at any age.

Regarding gonadal function, the timing of onset and the progression of pubertal attainment among individuals with DS are superimposable to those recorded in the general population. Nevertheless, in adult males compensated hypogonadism is a frequent finding and fertility is often compromised. On the other hand, women with DS display retained fertility, despite a slightly premature menopause.

From an auxological perspective, short stature is the main auxological outcome among individuals with DS. Though a potential impairment in the GHRH-GH-IGF-I axis has been hypothesized, the prescription of rhGH is still subject of debate, due to controversial data about its efficacy and safety in children with DS.

In addition, patients with DS are exposed to a four- and ten-fold increase in the risk of developing either type 1 or type 2 diabetes, respectively. Overweight children and adolescents with DS deserve a dedicated monitoring to timely detect and treat hyperglycemia.

Finally, young adults with DS tend to present a lower bone mineral density compared with age-and sex-matched healthy controls. Impaired osteoblast function and subsequent insufficient bone peak mass accrual during adolescence and young adulthood represent the key causative factors for low BMD in DS (120, 125, 127), rather than increased bone resorption. Accordingly, the potential role of antiresorptive agents is questionable in this population.

By adopting organ-specific health surveillance, from childhood to adulthood, an all-embracing care can be provided to individuals with DS and their families, addressing their specific needs and improving life expectancy and quality of life.

## Author contributions

SM: Conceptualization, Writing – original draft, Writing – review & editing. CF: Conceptualization, Writing – review & editing. MN: Writing – review & editing. SdM: Writing – original draft. MF: Writing – original draft. FL: Writing – original draft. LO: Writing – original draft. CP: Writing – original draft. ML: Writing – original draft. AG: Writing – review & editing. AL: Writing – review & editing. DS: Writing – review & editing. CV: Writing – review & editing. GC: Writing – review & editing. CD: Writing – review & editing. ABi: Writing – review & editing. ABa: Writing – review & editing. AC: Conceptualization, Writing – original draft, Writing – review & editing.
